# The *Families First* Program to Prevent Child Abuse: Results of a Cluster Randomized Controlled Trial in West Java, Indonesia

**DOI:** 10.1007/s11121-022-01433-w

**Published:** 2022-09-13

**Authors:** Mónica Ruiz-Casares, Brett D. Thombs, Nancy E. Mayo, Michelle Andrina, Susan C. Scott, Robert William Platt

**Affiliations:** 1 Department of Psychiatry, McGill University, Montreal, QC Canada; 2 SHERPA University Institute, CIUSSS du centre-ouest-de-l’île-de-Montréal, 7085 Hutchison, 204.2.14, Montreal, QC H3N 1Y9 Canada; 3 Lady Davis Institute for Medical Research, Jewish General Hospital, Montreal, QC Canada; 4 Research Institute, McGill University Health Centre, Montreal, QC Canada; 5 Department of Medicine and School of Physical and Occupational Therapy, McGill University, Montreal, QC Canada; 6 SMERU Research Institute, Jakarta, Indonesia; 7 Department of Epidemiology, Biostatistics and Occupational Health, McGill University, Montreal, QC Canada

**Keywords:** Parenting, Child maltreatment, Violence, Positive discipline, Randomized control trial, Indonesia

## Abstract

The *Families First* parenting program is a 10-week paraprofessional-administered adaptation of the Positive Discipline in Everyday Parenting program for West Java, Indonesia. It has not been tested in a randomized controlled trial. The objective was to evaluate the effects of *Families First* on physical and emotional punishment. We conducted a cluster randomized controlled trial and randomly assigned 20 rural and urban villages in West Java, Indonesia, to intervention or waitlist. Caregivers of children aged 0–7 years in intervention villages received *Families First*. Between 2017 and 2018, measurements were taken before randomization, immediately post-intervention, and 6 months post-intervention. Primary outcome was presence versus absence of *caregiver-reported physical or emotional punishment* immediately post-intervention*.* Intention-to-treat regression models accounted for clustering within villages and were run to compare between groups. Participants and study personnel could not be blinded. There were 374 caregivers in the 10 intervention villages and 362 in the 10 waitlist villages included in the trial and in outcome analyses. The intervention did not result in a lower proportion of intervention families using punishment immediately post-intervention (odds ratio [OR] for physical or emotional punishment immediately post intervention = 1.20 (95% CI 0.79–1.82). There were no significant differences for *positive and involved parenting, setting limits, and opinion on discipline*, but caregivers in the intervention group had significantly lower odds of using *positive discipline* (OR = 0.65 (95% CI 0.53–0.80). *Families First* did not prevent punishment in a setting with low levels of reported punishment but should be tested in a setting with higher levels or among people selected for risk or presence.

**Supplementary Information:**

The online version contains supplementary material available at 10.1007/s11121-022-01433-w.

## Introduction

Over half of all children globally experience physical and emotional violence, with high rates in low- and middle-income countries (LMIC) (Cuartas et al., 2019; Hillis et al., 2016), and most violence occurs in the context of punishment (Durrant & Ensom, 2012). The harmful consequences of early exposure to violence extend into adulthood and can result in premature mortality (Rogers et al., 2019) and significant health, economic, and social costs (Norman et al., 2012; Peterson et al., 2018; Widom et al., 2012). There is increasing interest in evidence-based early childhood home visiting and parenting programs to prevent and respond to violence against children (Easterbrooks et al., 2019; McCoy et al., 2020; Vlahovicova et al., 2017). However, there is concern about the applicability of existing evidence to LMIC (Mikton & Butchart, 2009), where little research originates (Coore Desai et al., 2017; Knerr et al., 2013) and very few preventive parenting programs combine group sessions with home visits (Hackworth et al., 2017). A review of parenting programs in Indonesia, with over 85 million children (UNICEF, 2019), of whom 39% of boys and 21% of girls have been reported to experience violence (Kerjasama et al., 2013), called for the testing of nonviolent discipline education for parents of young children (Tomlinson & Andina, 2015). The only previous randomized controlled trial (RCT) of a parenting program in Indonesia tested the Triple P program among 143 parents of children aged 2–12 years in Surabaya, East Java; it found greater reduction in the intervention group compared to waitlist in parent-reported children’s behavioral problems but not in emotional problems, the primary outcome. It did not include multiple facilitators or home visits (Sumargi et al., 2015).

We conducted a cluster RCT to assess the effect on child punishment of adding the *Families First* with Home Visitation Program (*Families First)* (Stewart-Tufescu et al., 2015) to standard community services. *Families First* is an adaptation to West Java, Indonesia, of the Positive Discipline in Everyday Parenting program. The program has been implemented in more than 25 countries, but no completed RCTs have tested its effects (Durrant, 2016, 2020). Anchored in children’s rights (Durrant & Stewart-Tufescu, 2017), it aims to offer parents a valid alternative to physical and emotional punishment, and to provide them with concrete conflict resolution tools, and information on children’s rights and development from birth through adolescence. It promotes strategies to strengthen parent–child communication, parents’ problem-solving skills, and emotional self-regulation of both parents and children. *Families First* added home visits and more group sessions to the program, adapted materials for cultural appropriateness, and translated them into Bahasa Indonesia. The program was pilot tested in 2015 and refined before this trial.

## Methods

### Study Design

This was a pragmatic, cluster RCT comparing effect on reducing child punishment of the *Families First* program added to standard government services provided by community health workers in West Java to standard services along in a waitlist control group. The protocol was approved by the Research Ethics Boards of the McGill Faculty of Medicine (Canada) and the Universitas Katolik Indonesia Atma Jaya (Indonesia) and followed closely. The trial was registered (NCT03374761) and the protocol was submitted for publication while the trial was ongoing (Ruiz-Casares et al., 2019). Trial registration was initiated before enrolment, yet only completed when data collection was underway due to staff changes and relocation of the trialist’s lab. No changes were made in trial procedures or outcomes between the time of trial initiation and registration and protocol submission.

There were amendments to the trial protocol. Measurement of the primary outcome was changed from an ordinal scale to a binary outcome due to the unexpectedly low number of participants with self-reported violence, which did not allow modeling an ordinal outcome. Additionally, since there was less than 3% missing data at the primary outcome assessment point and less than 4% 6 months later, missing values were replaced with the worst possible outcome (sensitivity analysis with best possible outcome for primary analysis) rather than using multiple imputation.

### Participants

The evaluation team together with program implementers selected 20 villages, among all villages in four Sub-Districts in Cianjur District, West Java, Indonesia, that had not previously had access to the program, were comparable in size, and were within driving distance from the district and sub-district capitals, and where local leaders had agreed to participation.

For caregiver recruitment, village authorities and service providers created initial lists of approximately 50 potentially eligible families in each village as per the study criteria listed below. The research team validated these lists for eligibility, then randomly selected a name to start inviting caregivers to participate and obtain written informed consent. To reach 36 participants per village, additional families were added to the list, if necessary. Eligible participants were female caregivers of children aged 0–7 years, who had at least one risk factor associated with the placement of children in residential care in Indonesia (i.e., living below the government poverty line or receiving social assistance, being a single or teenage mother, or having a father or mother who had migrated or a mother who is considering migration) (Puskapa Ui, 2014) as per the implementing agency’s child protection framework in Indonesia; resided in the village with no intention of moving in the coming year; spoke and read in Bahasa; had never participated in another parenting program; and had no cognitive impairment. One index child aged 0–7 was randomly selected per family. Socio-demographic characteristics at baseline are relayed in Table 1 and reveal that this was predominantly a low-income rural sample.

**Table 1:** Baseline characteristics

**Characteristics**	**Intervention (*n* = 374)**	**Waitlist (*n* = 362)**
**Household**		
Household size, mean (SD)	4.78 (1.70)	4.67 (1.59)
Number of children aged 0–7	1.30 (0.51)	1.25 (0.49)
Number of children aged 8–17	0.83 (0.89)	0.83 (0.89)
Presence of biological parents, *n* (%)		
Biological mother only	3 (0.80)	10 (2.76)
Biological mother and father only	77 (20.59)	54 (14.92)
Biological parents and others	249 (66.58)	253 (69.89)
Other	45 (12.03)	45 (12.43)
Number of adults caring for index child (excluding respondent), *n* (%)		
0	186 (49.73)	158 (43.65)
1	117 (31.28)	136 (37.57)
2	44 (11.76)	34 (9.39)
> 2	27 (7.22)	34 (9.39)
Electricity disconnected past year^a^, *n* (%)	11 (3.01)	5 (1.42)
Received any social security assistance at study enrollment^b^, %	209 (55.88)	176 (48.62)
Average household income ≤ 2 million Rupiah^c^, %	316 (84.95)	323 (89.23)
Rasch poverty location score, mean (SD)	0.64 (0.97)	0.40 (1.04)
Rasch parental conflict location score, mean (SD)	− 1.54 (1.08)	− 1.35 (1.22)
Age of biological mother, mean (SD), year	30.88 (7.33)	31.38 (6.97)
**Respondent/caregiver**		
Age at study enrollment, mean (SD), year	32.09 (8.78)	33.47 (9.12)
Marital status, *n* (%) married	362 (96.79)	342 (94.48)
Highest education degree^d^, *n* (%)		
Less than elementary school	9 (2.41)	17 (4.70)
Elementary school/A	218 (58.29)	201 (55.52)
Junior high school/B	112 (29.95)	97 (26.80)
Senior high/B and more	35 (9.36)	47 (12.98)
Main occupation: full-time homemaker, *n* (%)	318 (85.03)	315 (87.02)
Relationship to index child, *n* (%)		
Biological mother	349 (93.32)	327 (90.33)
Grandmother	20 (5.35)	29 (8.01)
Other	5 (1.34)	6 (1.66)
WHO-5 well-being (0–100), mean (SD)	64.67 (22.25)	64.61 (22.70)
Rasch Parental stress location score, mean (SD)	0.33 (1.20)	0.31 (1.19)
Rasch Perceived social support location score, mean (SD)	− 0.07 (1.33)	− 0.16 (1.24)
**Index child**		
Age at study enrollment, mean (SD), year	3.60 (2.05)	3.65 (1.99)
Sex, *n* (%) male	195 (52.14)	189 (52.21)
Attending pre-/school at study enrollment, *n* (%)		
Aged 0 to 4 years	17 (6.32)	26 (10.00)
Aged 5 to 7 years	81 (77.14)	91 (89.22)
Perceived good or excellent health, *n* (%)	311 (83.16)	302 (83.43)
Orphanhood (1 or both parents dead), *n* (%)	11 (2.94)	6 (1.66)
Developmental disability, *n* (%)	27 (7.22)	23 (6.35)
Family position, *n* (%)		
Eldest	69 (18.45)	65 (17.96)
Middle	36 (9.63)	23 (6.35)
Youngest	200 (53.48)	204 (56.35)
Only	69 (18.45)	70 (19.34)
Rasch stimulating environment location score, mean (SD)	− 0.93 (1.11)	− 0.92 (1.14)

^a^Among those with electricity

^b^The lower 40% of welfare group in Indonesia are entitled to receive social assistance including the national/local health insurance and the labor insurance (BPJS Ketenagakerjaan). In our sample, 50.95% of households received national/local health insurance and less than 2% received labor insurance, which covers mostly formal employment

^c^Minimum wage was around 1.8–2 million Rupiah per worker in 2016–2018 (West Java Province Gubernatorial Decrees No. 561/Kep. 1065, 1322, 1486)

^d^Elementary and junior high school include general and Islamic; senior high includes general, vocational, and Islamic

### Intervention

In each intervention village, three groups of approximately twelve caregivers received the *Families First* program consisting of 10 weekly face-to-face group sessions and four home visits of approximately 120 and 60 min each, respectively. Group sessions took place in community halls, early childhood centers, or, occasionally, in a facilitator’s home. Home visits included caregivers, their families, and neighbors. Catch-up sessions were arranged individually for participants unable to attend a session. The program was delivered between June and August 2017 by 60 locally recruited para-professional facilitators, who worked in pairs and were supervised on a weekly basis by one of 10 mentors. Facilitators and mentors were trained on the manualized program by Save the Children-Indonesia and two Master Trainers from the University of Manitoba. Through a series of interactive activities, parents were guided to identify their long-term childrearing goals, provide warmth and structure, understand how children aged 0–18 years think and feel, and problem-solve without punishment (Durrant, 2007, 2016, 2020). Additional information on *Families First*, including session content and profile of facilitators and mentors, is available in the study protocol (Ruiz-Casares et al., 2019). After data collection for the trial ended, the control group received the intervention between September and November 2018.

### Sample Size and Randomization

We estimated that a total of 720 caregivers would be needed to detect a decrease in the primary outcome of 15% for desired power of 0.90 with two-tailed *P* < 0.05, intra-class correlation of 0.02 (Murray & Blitstein, 2003), and 80% participation rate (Klar & Donner, 2001).

After baseline assessments and immediately preceding delivery of the intervention, we randomized villages stratified by urban/rural location at a 1:1 ratio for intervention and waitlist arms. To maintain both local and scientific trust in the allocation process, the research team led a video-recorded randomization ceremony attended by all the village chiefs and staff from the implementing agency. The structured lottery and documentation procedure used 20 sequentially numbered (1–7 “Urban” and 1–13 “Rural”), opaque, lined with aluminum foil, sealed, and stapled envelopes independently prepared by the first author. Envelopes were drawn by urban villages, followed by rural villages.

### Data Collection Procedures

Data were collected at three time points: enrollment, immediately post-intervention (21 weeks post enrollment), and 6 months post-intervention (46 weeks post-enrollment). All measures were independently forward- and back-translated (Bahasa and Sundanese), pilot tested, and programmed into the Census and Survey Processing System, a public domain software package for collecting survey data. All measures except for program satisfaction (only assessed immediately post-intervention to intervention participants) and most socio-demographic items were administered at the three measurement points. Tablet-based questionnaires were administered orally by female university graduates fluent in Bahasa or in Sundanese in caregivers’ homes. Administrators were not blind to group allocation but were trained on consistent administration of measures, awareness of biases, and ethical conduct of research prior to each data collection point. Implementers and researchers in Indonesia followed Save the Children’s Child Safeguarding Policy. Caregivers were informed during the consent process of the need to report situations that indicate significant risk of harm to a child and to connect caregivers to appropriate services. No severe cases of abuse were observed. No financial incentives were provided for participation in the evaluation; only a small token of appreciation (e.g., a small pouch) was given after each interview. The implementing partner only provided a branded T-shirt to acknowledge completion of the parenting sessions.

Process evaluation documented attendance, engagement, implementation fidelity, and satisfaction through facilitator-completed tracking forms, independent observations, and post-intervention questionnaire and individual and group interviews with caregivers and implementers. However, since facilitator records were often incomplete or suggested implausible patterns, only results from the post-intervention survey of caregivers were used. A qualitative assessment of caregiver, facilitator, and mentor experiences will be reported elsewhere.

### Outcomes

#### Primary Outcome

The primary outcome was absence versus presence of caregiver reported physical or emotional punishment. It was measured with three self-report items (one each on severe physical abuse, moderate physical abuse, and emotional abuse) inspired by the International Society for Prevention of Child Abuse and Neglect Child Abuse Screening Tool (ICAST-Parent) (Runyan et al., 2009) inquiring how many times had caregivers used these types of punishment with the index child in the past month and ever. Due to unexpected low levels of self-reported violence in our sample, this required adjusting the measurement from an ordinal scale, as originally planned, to a binary outcome, with no physical or emotional punishment compared to any, defined as a positive response on any of the three items.

#### Secondary Outcomes

Secondary outcomes included explanatory outcomes that address mechanisms by which patient outcomes may be achieved, and exploratory outcomes, which are downstream outcomes that follow the hypothesized effect (Fairclough, 2002; Mayo et al., 2016). Information on the theoretical model and study measures can be found in the study protocol (Ruiz-Casares et al., 2019) and in Online Resource 1. Explanatory outcomes included (a) positive and involved parenting (analyzed separately because items did not fit a Rasch model (Online resource 1); 6 items adapted from the Alabama Parenting Questionnaire (APQ) (Essau et al., 2006); one item from the Positive Parenting Subscale (praise house help) was excluded from the overall score as it only applied to 5–7-year-olds); (b) positive discipline (4 items from the ICAST (Runyan et al., 2009); (c) setting limits (2 items from the Parenting Young Children (PARYC), analyzed separately as they did not fit the Rasch model (McEachern et al., 2012)); and (d) opinion on discipline (2 items from the ICAST-Parent (Runyan et al., 2009)).

Exploratory outcomes, downstream outcomes that follow the hypothesized effect, were (a) *child social and emotional wellbeing* (total difficulties score calculated from the emotional, conduct problems, hyperactivity, and peer problems scales of the Strengths and Difficulties Questionnaire parent versions for children aged 2 years and older) (Goodman, 1997)); (b) *attitudes towards institutionalization of children* (4 items adapted from the Child Protection Knowledge, Attitudes, and Practices (Ruiz-Casares, 2011) and 4 new items); and (c) *monitoring/supervision* (10 items from the APQ and 1 item from the Parent Supervision Attributes Profile Questionnaire (Morrongiello & Corbett, 2006); scores were calculated separately for children aged 0–4 and 5–7 years, since item wording differed by age group).

#### Contextual and Other Influencing Factors

Contextual and other influencing factors in parenting and child abuse (Table 1) were considered potential confounders and included *parenting stress* (18 items from the Parental Stress Scale) (Berry & Jones, 1995); *caregiver mental health* (5 items from WHO Wellbeing Index) (Topp et al., 2015); *perceived social support* (8 items adapted from the modified Medical Outcomes Study Social Support Survey) (Sherbourne & Stewart, 1991); *stimulation in the home environment* (6 items from the Multiple Indicator Cluster Survey) (UNICEF, 2013), one item adapted from the APQ, and two additional items (sharing meals and exploring toys alone); and *inter-parental conflict regarding child-rearing* (11 items from the Parent Problem Checklist (Morawska & Thompson, 2009).

### Statistical analyses

Analyses were conducted using an intent-to-treat (ITT) approach and performed with SAS^®^ Version 9.4 for Windows. Baseline characteristics of caregivers in the two groups were reported using frequency distributions and descriptive statistics. Logistic and linear regression models with generalized estimating equations to adjust for randomization by village were run using the SAS genmod procedure to compare between groups immediately post-intervention for binary and normally distributed continuous outcomes, respectively. When continuous outcomes were not normally distributed, they were categorized and analyzed with a proportional odds model via the SAS glimmix procedure to account for clustering by village. Homogeneity was examined in the ordinal models. When data were too sparse or the model did not converge, ordinal outcomes were collapsed and modeled as binary outcomes. The estimates for all models are presented with their 95% confidence intervals (CIs) and were run in the direction of a better outcome, unless otherwise indicated. The outcome at baseline, socio-economic status (SES), child’s age, and stimulating environment at baseline were included as potential confounders of the primary outcome only. No other variables showed large imbalances by randomization group and were added to this model.

Pre-specified sub-group analyses were run with first-time parents, mothers who were under 20 years of age at the birth of the index child, families with more children or children of different age groups, and by sex, and disability status of the child. Secondary analyses estimated the impact of the program on the other relevant outcomes. Exploratory analyses were conducted to compare caregivers who had completed the core package as defined by the implementing agency (i.e., group sessions 2, 3, 7, 9, and 10 and any one home visit) and those who had not, as well as among those with and without violence reported at baseline. Results for 6 months post-intervention were only examined as exploratory analyses. Unadjusted results for these secondary and exploratory analyses are presented. For subjects without immediately or 6-months post-intervention data, missing binary outcomes were replaced with the worst possible value and continuous outcomes were assigned a poor but not the most extreme value so as not to disrupt the tail of the distribution. The primary analysis (unadjusted) was also run with the best possible outcome. For subgroup analyses, whenever updated socio-demographic information was not available, subgroups were formed using status from previous visits. Descriptive statistics were used to explore attendance. Statistical significance was established at an alpha level of 0.05; no corrections for multiple comparisons were done.

## Results

**Fig. 1:**
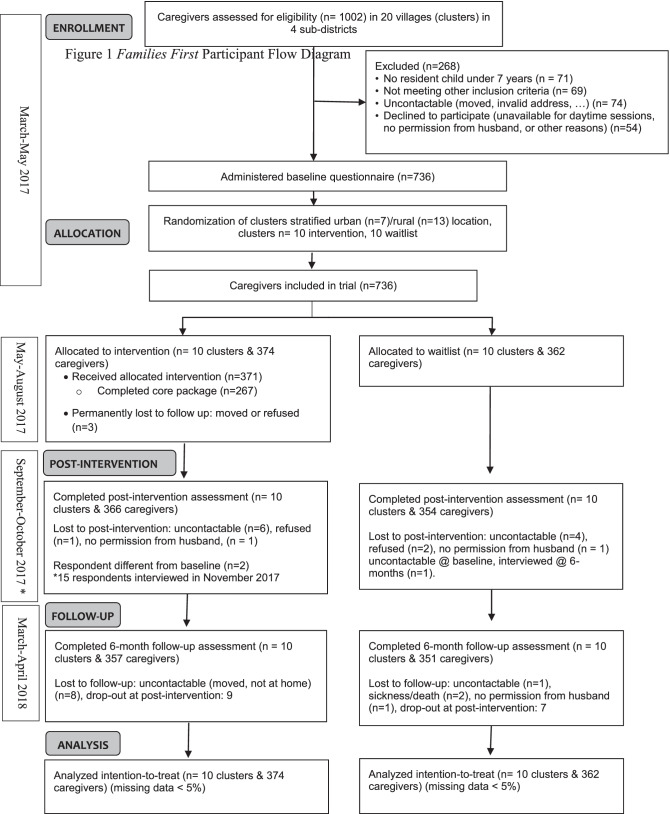
*Families First* participant flow diagram

A total of 1002 caregivers were screened in 20 villages and 736 met eligibility criteria and were randomized in May 2017 (Fig. 1). A mean of 36.8 families (SD 1.1, range 36–40) per village and a total of 374 and 362 caregivers were allocated to the intervention and the waitlist arms respectively. Participant loss after randomization was low: 371 caregivers received the program as randomized and 3 dropped out due to relocation or refusal to participate. Eight caregivers were lost to follow-up immediately post-intervention in each arm due to inability to locate or refusal (participant or husband). At 6 months post-intervention, losses to follow-up (17 intervention, 11 waitlist) were due to inability to locate, sickness, lack of permission from husband, and earlier drop-out. Table 1 lists baseline characteristics of the intervention and waitlist groups.

### Program Delivery

On average, caregivers in the intervention group received 9.0 group sessions (SD = 1.8; median = 10) and 3.7 home visits (SD = 1.0; median = 4) over the course of 3 months. Overall, 5.6% of the 374 caregivers did not receive any home visits, 7.5% of caregivers in the intervention group missed at least 2 of the 5 core group sessions, and 71.4% complied to the core package. In 61.0% of the 366 cases interviewed post-intervention reporting any missed group session or home visit, either a facilitator (37.2%) or another caregiver (23.8%) explained the material to the absent caregiver. However, it was not possible to know what specific sessions were recuperated.

### Program Effect

Tables 2 and 3 show program effects immediately post-intervention. At post-intervention, the primary analysis (unadjusted) revealed that although the intervention group (280/374 = 74.9% with no reported violence immediately post-intervention, including the eight lost families who were assigned an outcome of violence) had higher odds of reporting no physical or emotional punishment than the waitlist group (258/362 = 71.3% reporting no violence immediately post-intervention, including the eight lost families assigned an outcome of violence), this effect was not statistically significant (OR for no harsh punishment immediately post intervention = 1.20; 95% CI: 0.79, 1.82; *p* = 0.39) (Table 2). These results remained consistent when the model was adjusted for baseline violence and other potential confounders, when the best-case substitution was used (data not shown), or when only complete cases were considered (Table 3). Pre-specified subgroup analyses did not document any significant effects between intervention and waitlist groups immediately post-intervention or at 6 months post-intervention (data not shown for the latter).

**Table 2:** Regression models of intent-to-treat program effects at post-intervention

		***n***	**OR/POR/Beta**^a^	**95% CI**	***P***-**value**
**Primary outcome** *(users vs. non-users of harsh punishment)*					
**Unadjusted**					
**Primary analysis**		736	1.20	0.79, 1.82	0.387
Subgroup analysis					
Sex index child					
Male		384	1.31	0.81, 2.11	0.266
Female		352	1.08	0.61, 1.92	0.795
Position of index child at end of the intervention					
Eldest child		140	1.32	0.59, 2.95	0.495
Only child		133	0.82	0.37, 1.82	0.626
Developmental disability reported at/prior to end of the intervention		71	2.92	0.60, 14.14	0.184
Number of children in household					
Maximum of 2		539	1.11	0.71, 1.73	0.634
More than 2		197	1.34	0.76, 2.36	0.304
More than 3		64	1.58	0.51, 4.88	0.427
Families with children in both age groups (0–7 and 8–18 years)		417	1.18	0.75, 1.87	0.479
Biological mother < 20 years old					
At birth of child		122	1.10	0.54, 2.25	0.800
At intervention		9			
ICC ^b^	0.029			0, 0.062	
**Adjusted for baseline, age of index child, household SES, stimulating environment**		736	1.12	0.75, 1.68	0.585
**Secondary outcomes (unadjusted)**					
**Positive parenting: POR**		736	1.30	0.86, 1.95	0.213
**Involved parenting (age > 5): OR**		158	2.23	0.73, 6.80	0.158
**Positive discipline: OR**		736	0.65	0.53, 0.80	< 0.001
**Setting limits**					
** Sticking to rules: OR**		736	0.74	0.53, 1.02	0.069
** Speaking calmly when upset: OR**		736	1.07	0.65, 1.77	0.798
**Opinion on discipline: OR**		736	0.88	0.52, 1.48	0.619
**Child’s social and emotional well-being (total difficulties), ages ≥ 2**^c^		580	*b* = 0.90	− 0.01, 1.80	0.052
**Attitudes towards the institutionalization of children**^d^		736	*b* = 0.05	− 0.17, 0.26	0.657
**Monitoring/supervision, ages 0 to 4**		483	POR = 1.04	0.68, 1.59	0.860
**Monitoring/supervision, ages 5 to 7**		253	POR = 0.97	0.62, 1.53	0.904
**Post hoc subgroup analysis of primary outcome post-intervention by baseline punishment status and model (OR no punishment)**					
**Non-users of punishment**	Unadjusted	471	0.95	0.54, 1.67	0.865
	Adjusted^e^		0.80	0.44, 1.47	0.475
**Users of punishment**	Unadjusted	265	1.75	1.10, 2.78	0.019
	Adjusted^e^		1.70	1.09, 2.66	0.019

The eight families lost in each arm were consistently assigned the worst outcome

^a^Odds ratio (OR) for logistic regression (primary outcome: no punishment; explanatory outcome: better outcome); proportional odds ratio (POR) for ordinal regression, beta (b) for linear regression (Exploratory outcome: better outcome unless otherwise specified)

^b^Intracluster correlation coefficient (ICC) at immediately post-intervention visit

^c^This is a score of difficulties; hence, a higher score and a positive beta indicate a worse outcome in the intervention group

^d^Higher is less pro-institutionalization. Score obtained using Rasch analysis

^e^Adjusted for age of the child; stimulating environment; household SES; and level of punishment at baseline (for users of physical and emotional punishment at baseline)

**Table 3:** Outcome data at baseline and immediately post-intervention (complete data only)

**Outcome**	**Baseline**								**Post-intervention**								
	Intervention				Waitlist				Intervention					Waitlist			
	*N*	*N*/mean		%/SD	*N*	*N*/mean		%/SD	*N*		*N*/mean		%/SD	*N*	*N*/mean		%/SD
**Primary outcome**																	
Harsh punishment: none (v any)	374		232	62.0%	362	239		66.0%	366	280			76.5%	354	258		72.9%
**Secondary outcomes**																	
**Positive parenting** (items scored in 2 best levels)	374				362				366					354			
0: all items			153	40.9%		117		32.3%		122			33.3%		95		26.8%
1: 4 items			161	43.0%		179		49.4%		177			48.4%		181		51.1%
2: 3 items			36	9.6%		42		11.6%		42			11.5%		53		15.0%
3: < 3 items			24	6.4%		24		6.6%		25			6.8%		25		7.1%
**Involved parenting** ages > 5 years, 1–2 (v 0 parents)	62		53	85.5%	57	46		80.7%	80	72			90.0%	76	56		73.7%
**Positive discipline** 3 best levels (v 2 worst)	374		147	39.3%	362	137		37.8%	366	214			58.5%	354	169		47.7%
**Setting limits**: more often (v never/almost never)																	
*Sticking to rules*	374		320	85.6%	362	321		88.7%	366	296			80.9%	354	303		85.6%
*Speaking calmly when upset*	374		357	95.5%	362	346		95.6%	366	336			91.8%	354	323		91.2%
**Opinion on discipline** corporal punishment never effective (v more effective)	374		341	91.2%	362	323		89.2%	366	331			90.4%	354	325		91.8%
**Child’s social and emotional well-being**^a^ (total difficulties), ages ≥ 2 years	258		10.28	4.50	268	10.20		4.83	282	10.85			4.44	289	9.96		4.33
**Attitudes towards institutionalization**^b^	374		− 0.42	1.07	362	− 0.39		1.02	366	− 0.34			1.22	354	− 0.39		1.03
**Monitoring/supervision**																	
*Ages 0 to 4 years*	264				260				239					231			
0: best			136	51.5%			138	53.1%				112	46.9%			106	45.9%
1			17	6.4%			12	4.6%				17	7.1%			19	8.2%
2			57	21.6%			42	16.2%				53	22.2%			49	21.2%
3			13	4.9%			19	7.3%				14	5.9%			13	5.6%
4			23	8.7%			23	8.8%				16	6.7%			22	9.5%
5: worst			18	6.8%			26	10%				27	11.3%			22	9.5%
*Ages* ≥ *5 years*	110				99				127					123			
0: best			18	16.4%			23	23.2%				20	15.7%			28	22.8%
1			30	27.3%			18	18.2%				26	20.5%			20	16.3%
2			9	8.2%			16	16.2%				26	20.5%			18	14.6%
3			24	21.8%			25	25.3%				30	23.6%			24	19.5%
4			19	17.3%			5	5.0%				13	10.2%			24	19.5%
5: worst			10	9.1%			12	12.1%				12	9.5%			9	7.3%

^a^The score for total difficulties in child’s well-being was on average 0.9 points worse in the intervention group (*p* = 0.052)

^b^Higher is less pro-institutionalization

Some evidence of an effect was observed among users who reported physical and emotional punishment at baseline (OR for reduction to no violence = 1.75; 95% CI: 1.10, 2.78; 90/142 = 63.4% in the intervention group including two lost who were assigned an outcome of punishment and 61/123 = 49.6% in the waitlist group including three lost who were assigned an outcome of punishment, *p* = 0.02), even after adjusting for confounders (Table 2), and among caregivers in the intervention group who received the core package (*n* = 267) compared to those who did not (*n* = 107) (unadjusted OR 1.91 (95% CI 1.26, 2.89, *p* = 0.002)) (data not shown).

Immediately post-intervention, caregivers in the intervention group had significantly lower odds of using *positive discipline* than the waitlist group (OR = 0.65 (0.53, 0.80)). There were no significant differences between groups immediately post-intervention in any of the other secondary outcomes (*positive and involved parenting*, *setting limits*, *opinion on discipline*, *children’s social and emotional well-being*, *attitudes towards the institutionalization of children*, and *monitoring/supervision*).

## Discussion

This first RCT of *Families First* found that the program did not prevent physical and emotional punishment of children immediately post intervention. There were no significant differences for *positive and involved parenting*, *setting limits*, *and opinion on discipline*, but caregivers in the intervention group had significantly lower odds of using *positive discipline* immediately post-intervention.

Reduced punishment immediately post-intervention specifically among users of punishment at baseline and among caregivers who received the core package holds promise, yet should be considered with caution as these were unplanned, post hoc analyses, therefore requiring validation in a new trial. The low punishment level in the study sample, the need to adapt the primary outcome analysis, and the lack of evidence for an effect on the primary outcome in this population do not preclude the possibility that the intervention would be effective in reducing punishment in a different population or for a group with full exposure to the core sessions.

We did not find any statistically significant differences in the pre-specified subgroup analyses, or in secondary outcomes except for decreased positive discipline immediately post-intervention. These results are potentially susceptible to multiple testing and should be validated in a follow-up study. Further research will also be needed to explore determinants of caregivers’ ability to reflect and report on their own parenting behaviors, including their level of understanding of positive discipline. Although the program was deemed to be culturally appropriate by caregivers and facilitators, our results regarding violent and positive discipline practices may call for program revision as promoting positive discipline strategies will likely be insufficient to eliminate all forms of violent discipline (Cuartas et al., 2019).

Several limitations must be considered in interpreting the results. First, unexpected low levels of self-reported violence in the sample at baseline required that a binary outcome be used rather than the initially proposed continuous scale. Hence, no conclusion can be reached about the effectiveness of the program to produce gradual changes in use of harsh punishment. Our findings may underestimate program effectiveness in higher-risk populations and may reflect the possibility that the study sample is not an appropriate target for the intervention. Piloting and testing of trial procedures and locally developed or adapted measures in similar communities and access to reliable epidemiological data disaggregated geographically will be crucial to inform future studies and programs.

Second, response bias must be considered in self-report measures, particularly on sensitive issues (e.g., harsh punishment and abuse) and in the context of strong sociocultural norms (e.g., it is considered rude to criticize a free service). The use of self-reports common in parenting programs was necessary in the absence of official records of child maltreatment. Interviews were conducted in private to improve reliability of reporting. No firm conclusions could be drawn about exposure because attendance was self-reported by caregivers and inadequately recorded by facilitators in monitoring forms. The same recording limitation applies to fidelity; few observations of group sessions were conducted due to scheduling and material constraints, and concerns over interference prevented observations of the home visits.

Third, blinding of interviewers and participants is very hard to maintain in village-level cluster trials. To avoid preferential behavior towards the intervention group, the interviewers were trained in the standardized administration of measures and awareness of personal biases. Non-blinded outcome assessment can lead to overestimation of intervention benefits, but his was not likely an issue in our trial since we did not find that the intervention improved targeted outcomes. Fourth, due to fear of cross-contamination and interference, implementing agency staff took significant distance from the villages throughout the trial. This included delegating the preparation of the initial lists of participants from which researchers sought consent to participate to local authorities and service providers. The ensuing confusion among some participants regarding the roles of implementers and researchers may have influenced caregivers’ answers.

Fifth, pilot testing of standardized outcome measures in Cianjur District resulted in adaptation of some measures and substitution of others for particular indicators, thus hindering comparisons across studies. Further testing and adaptation may be needed (e.g., it is unclear whether the outcome *setting limits* is adequately captured by the two items considering the borderline significance of one and the non-significance of the other). Limited applicability of items or measures for certain ages hindered comparisons across age groups. Several items did not fit a Rasch model to create a continuous unidimensional measure.

Sixth, timing of post-intervention measurements was limited to immediately after program delivery and 6 months later. Partial overlap of data collection and recall period immediately post-intervention may have further limited results. Seventh, it was not possible to ascertain individual exposure to other social programs in all villages. Nonetheless, visits to waitlist villages prior to all data collection times did not identify new parenting programs. Eighth, despite the use of pragmatic trial methods, given the limited number of clusters in one district in West Java and the homogeneity of the sample in terms of sex, religion, and language, it is uncertain the extent to which our results are generalizable to other individuals, populations, or settings. Finally, outcomes were not measured from fathers or other home visit participants.

## Conclusion

This study makes an important contribution to the literature on evidence-based, culturally adapted parenting programs to reduce violence against children in LMIC, and specially to policy and delivery of this intervention at a global scale. It provides accurate estimates of the effect of the program for participating caregivers immediately post-intervention and 6 months later. Null effects for the primary (*physical and emotional punishment*) and key secondary outcomes (*positive and involved parenting*, *setting limits*, and *opinion on discipline*) call for reconsideration of program targeting and content and study measures and indicators. At the same time, improved self-reported positive discipline provide a good basis for further research on the effectiveness of the program. The trial was conducted independently from program developers, measured reported acts of violent discipline and opinions about punishment in a large sample, and used strong pragmatic cluster randomized methods—including a rigorous and culturally appropriate random allocation process, and ITT analyses. The trial also paid close attention to ethical issues and strengthened capacity by recruiting and training local researchers. The program was delivered by trained non-professionals in community settings. There was very low loss to follow-up, which has also been documented in other similar trials in Indonesia (Sumargi et al., 2015) and other LMIC (Lachman et al., 2017; Puffer et al., 2015). Open collaboration across partners and with a local advisory committee contributed to the cultural appropriateness of research procedures, high recruitment and retention rates, and the relevance of the study for policy and programming.

With 1 billion children globally experiencing violence every year, violence prevention constitutes a major public health issue (Hillis et al., 2016). Violence against children can have long-term consequences on a child’s physical, brain, and psycho-social development (Norman et al., 2012; Widom et al., 2012) and in the social and economic fabric of societies (Peterson et al., 2018). The highest prevalence in LMIC demands increased attention to the creation, adaptation, and evaluation of locally appropriate prevention and intervention programs. Parenting programs should be evaluated both for reducing violent discipline and for increasing positive discipline.

## Supplementary Information

Below is the link to the electronic supplementary material.

Supplementary file1 (DOCX 38 KB)

Supplementary file2 (DOCX 31 KB)
