# Supplementary material for: The Families First Program to Prevent Child Abuse: Results of a Cluster Randomized Controlled Trial in West Java, Indonesia
Source: Prev Sci. 2022 Sep 13;23(8):1457–69. doi: 10.1007/s11121-022-01433-w (PMC9587081; doi:10.1007/s11121-022-01433-w)
Supplement: Supplementary file 1 — Supplementary file1 (DOCX 38 KB) [file 11121_2022_1433_MOESM1_ESM.docx]

**Online Resource 1 – *Families First* outcome measures as analyzed**

| **Outcome** | **Source** | **Analysis adjustments** | **Rationale** |
| --- | --- | --- | --- |
| **Primary outcome** | | | |
| **Frequency of use of physical and emotional punishment** | 3 items inspired by the International Society for Prevention of Child Abuse and Neglect Child Abuse Screening Tool (ICAST-Parent) (Runyan et al., 2009) | Measurement adjusted from an ordinal scale to a binary outcome (i.e., any frequency on any of the three items vs never on all three). | Unexpected low levels of self-reported violence in sample |
| **Secondary Outcomes (Explanatory outcomes)** | | | |
| **Positive and involved parenting** ^a^ | 6 items adapted from the Positive Parenting Subscale and 1 item from the Involvement Subscale of the Alabama Parenting Questionnaire (APQ)  (Essau et al., 2006) | Analyzed separately positive and involved parenting.  One item from the Positive Parenting Subscale (praise house help) was excluded from the overall score.  Count (0-5) of 2 best levels for 5 items | Items did not fit the Rasch model (McEachern et al., 2012).  Involved parenting item only applied to >5-year-olds. |
| **Positive discipline** ^a^ | 4 items from the Nonviolent Subscale of the ICAST (Runyan et al., 2009) | Three best levels (scores 0-2) vs two worst levels (scores 3-4) where the 0-4 score was created by summing dichotomies of all 4 items based on cuts found from Rasch. | Items did not fit the Rasch model. |
| **Setting Limits** ^a^ | 2 items from the Setting Limits Subscale of the Parenting Young Children (PARYC) | Items analyzed separately.  For each, model  3 best levels vs 2 worst (never/almost never) | Items did not fit the Rasch model. |
| **Opinion on discipline** | 2 items from the ICAST-Parent (Runyan et al., 2009) | First item analyzed qualitatively as planned. Second item dichotomized at “never effective” vs the other three. | Distribution of responses (90% of were “never effective”). |
| **SECONDARY outcomes (Exploratory outcomes)** | | | |
| **Child social and emotional wellbeing** | Strengths and Difficulties Questionnaire (SDQ) parent versions for 2-3 year-olds and 4-17 year-olds (23) (Goodman, 1997). Total Difficulties score excludes prosocial scale (i.e., Helpful if someone is hurt, Kind to younger children, and Often volunteers to help others). |  |  |
| **Attitudes towards institutionalization of children** ^a^ | 4 items adapted from the Child Protection Knowledge, Attitudes, and Practices (CP-KAP) (Ruiz-Casares, 2011) and 4 new items | A continuous score was produced using Rasch analysis. |  |
| **Monitoring/**  **Supervision** | 10 items from the Poor Monitoring/Supervision scale of the APQ and 1 item from the Parent Supervision Attributes Profile Questionnaire (PSAPQ) (Morrongiello & Corbett, 2006) | Scores calculated separately for children aged 0-4 and 5-7 years | Item wording differed by age group. |
| **other influencing factor in parenting and child abuse USED FOR ADJUSTMENT** | | | |
| **Stimulation in the home environment** ^a^ | 6 items from the Early Childhood Development Module of the Multiple Indicator Cluster Survey (UNICEF, 2013), 1 item adapted from the Involvement Subscale of the APQ, and 2 additional items (sharing meals and exploring toys alone). | A continuous score was produced using Rasch analysis, allowing for different scoring for questions worded differently by age. | Item wording differed by age group. |

*Note:* _a_ Intended to model as a Rasch score as per protocol.
